# Long-Term Transcranial Direct Current Stimulation Does Not Improve Executive Function in Healthy Older Adults

**DOI:** 10.3389/fnagi.2018.00298

**Published:** 2018-10-17

**Authors:** Lijuan Huo, Zhiwei Zheng, Jin Li, Wenyu Wan, Xiaoyu Cui, Shuyuan Chen, Wei Wang, Juan Li

**Affiliations:** ^1^Center on Aging Psychology, CAS Key Laboratory of Mental Health, Institute of Psychology, Chinese Academy of Sciences, Beijing, China; ^2^Department of Psychology, University of Chinese Academy of Sciences, Beijing, China

**Keywords:** transcranial direct current stimulation, executive function, dorsolateral prefrontal cortex, older adults, follow-up effect

## Abstract

**Background**: Executive function tends to decline as people age. Transcranial direct current stimulation (tDCS) is assumed to have beneficial effects on various cognitive functions. Some prior investigations have shown that repeated sessions of tDCS enhance the executive function performance of healthy elderly people by mediating cognitive training gains. However, studies of the effect of long-term stimulation on executive function without cognitive training are absent.

**Objective**: The purpose of this study was to explore whether the executive function of healthy older adults could be enhanced with long-term tDCS alone applied on the prefrontal cortex.

**Methods**: Sixty-five cognitively normal older adults were enrolled and randomly assigned to two groups: an anodal tDCS group and a sham tDCS group. The participants in the two groups received anodal stimulation or sham stimulation over the left dorsolateral prefrontal lobe, for 30 min per day for 10 consecutive days. Executive function was tested before stimulation, immediately after stimulation and 3 months after stimulation. Three core components of executive function were tested using a two-back task for updating, a flanker task for inhibition, and a switching task for shifting.

**Results**: Across the three tasks, we failed to discover any differences between the anodal and sham stimulation. Moreover, we found no statistically significant stimulation effect in the follow-up session.

**Conclusion**: Our study does not support the assumption that multiple sessions of tDCS that are independent of cognitive training have a beneficial effect on executive function in healthy older adults, presumably because the effect of the stimulation lies in its amplification of training gains. It indicates that combining traditional cognitive training methods with brain stimulation may be a better approach to improve older adults’ executive function.

## Introduction

Executive function is loosely defined as a set of higher-level cognitive abilities that are involved in coordinating various cognitive resources in order to complete complex cognitive tasks (Diamond, 2013). Miyake et al. (2000) summarized a number of executive function tasks and identified three core subcomponents, which are widely recognized and extensively used: updating or working memory, inhibition and shifting or cognitive flexibility. Updating describes the capacity to dynamically modify and replace information in the brain based on new information; this is typically measured by running span or n-back tasks. Inhibition describes the ability to suppress irrelevant or interfering information, and to respond to target stimuli as soon as possible in many inhibitory tasks, such as the flanker task, stop signal task, go-no go task and Stroop task. Shifting refers to the ability to flexibly switch between tasks or demands of attention, and its common paradigms are switching tasks and dual tasks.

The prefrontal lobe, especially the dorsolateral prefrontal cortex (DLPFC), is the most important neural basis of executive function (Alvarez and Emory, 2006; Yuan and Raz, 2014). Traditionally, neuropsychological tests of executive function have been widely used to measure frontal lobe function (Alvarez and Emory, 2006). However, with the development of neuroimaging technologies, there is mounting evidence that executive function is supported by the DLPFC (Brodmann’s areas, BA 9 and BA 46; e.g., Alvarez and Emory, 2006; Anderson et al., 2008; Niendam et al., 2012; Fuster, 2013). The critical components of executive function consistently share a common pattern of DLPFC activation. The DLPFC is involved in general executive processes (Collette et al., 2006; Yuan and Raz, 2014), including updating (Curtis and D’Esposito, 2003), inhibition (Durston et al., 2003) and shifting (Wager et al., 2004; Witt and Stevens, 2013). Methods of neurostimulation (e.g., transcranial magnetic stimulation) have confirmed the causal relationship between the DLPFC and updating, inhibition and shifting (Mull and Seyal, 2001; Vanderhasselt et al., 2006; Kim et al., 2012; Brunoni and Vanderhasselt, 2014). This crucial region provides top-down cognitive control and generally plays a role in the maintenance, monitoring and temporal organization of information (Duncan and Owen, 2000; MacDonald et al., 2000; Koechlin et al., 2003; Ridderinkhof et al., 2004).

With the atrophy and deterioration of the prefrontal lobe, executive dysfunction is prevalent among healthy seniors, and it mediates and explains other age-related cognitive decline (Salthouse et al., 2003; Crawford et al., 2000; Turner and Spreng, 2012). As a primary symptom of neurodegenerative diseases, executive dysfunction has become a pressing issue of cognitive aging. As such, effective intervention to prevent and delay its decline would greatly benefit both individuals and society.

More recently, transcranial direct current stimulation (tDCS) has drawn substantial attention for its potential to improve cognitive function. tDCS provides a non-invasive means of modulating cortical plasticity by delivering a weak direct electrical current (0.5–2 mA) with at least two surface electrodes on the scalp. Stimulation increases cortical excitability via the anodal electrodes and decreases cortical excitability via the cathodal electrodes. There is plenty of evidence that tDCS enhances cognitive performance and boosts cognitive training gains (e.g., Jacobson et al., 2012; Bourzac, 2016; Dedoncker et al., 2016). Consistently, tDCS seems to be a promising tool for slowing down the deterioration of the executive function of elderly people.

Regarding its neurobiological mechanisms, anodal tDCS (atDCS) increases neuronal excitability, making the neurons more ready to fire in reaction to the information input, or it induces long-term potentiation (LTP)-like processes, modifying synaptic strength and facilitating synaptic communication (Bennabi et al., 2014; Medeiros et al., 2012; Prehn and Flöel, 2015). Therefore, it is assumed that cognitive function can be modulated by anodal stimulation of the specific brain region involved in the function. In the current study, the stimulation site would be DLPFC, the core region of executive function. The beneficial effect may be cumulatively gained and it may be gained by repeated stimulation, inducing prolonged neuronal excitability and enhancing synaptic plasticity (Monte-Silva et al., 2013; Bastani and Jaberzadeh, 2014).

Multiple sessions of tDCS possibly have a more favorable effect than a single session of tDCS (Boggio et al., 2012; Khedr et al., 2014; Horvath et al., 2015; Hsu et al., 2015a; Savic et al., 2017). Existing studies found that single-session stimulation may be not substantial enough to induce a reliable effect (Horvath et al., 2015; Savic et al., 2017). In addition, multiple sessions of tDCS could produce cumulative gains over time, so that it induced long-term aftereffects more easily (Boggio et al., 2012; Khedr et al., 2014). The idea was also proved by meta-analytic evidence with older adults. Hsu et al. (2015a) summarized quantitative data, showing that the effect size of studies with multiple sessions is much larger (ES = 0.89) than that with single sessions (ES = 0.44).

Nevertheless, the effect of long-term stimulation alone on the executive function components of updating, inhibition, and shifting in elderly people has not been detected so far. There has been a limited number of previous studies, which mainly focused on working memory updating. These studies usually combined tDCS with working memory updating training, aiming to enhance working memory by amplifying training gains (Park et al., 2014; Jones et al., 2015; Stephens and Berryhill, 2016; Nilsson et al., 2017). However, some constraints of cognitive training, such as the boring repeated practice and difficulties with learning new strategies, have caused problems with the compliance of older adults and a high cost of training. As such, using tDCS to modulate executive function performance without cognitive training has advantages. Thus, studies in which participants receive tDCS without working memory training are needed to clarify whether tDCS alone provides benefits or whether the combination of tDCS and training does.

Therefore, the aim of the current study was to investigate whether repeated sessions of tDCS alone can counter age-related executive dysfunction. To address this question, participants received anodal or sham tDCS above the left DLPFC for 10 consecutive days. Before and after the treatment, they completed three tasks that involved updating, shifting and inhibition. In addition, some studies have found that positive results are not obtained until a certain period of time has passed (Doruk et al., 2014; Biundo et al., 2015; Jones et al., 2015). For instance, in the study of Biundo et al. (2015), the performance on the cognitive tests had improved only at a 16-week follow-up. To evaluate the possible delayed effect and long lasting effect of tDCS, executive function was assessed again 3 months later. As previously described, the DLPFC is the most crucial site for updating, shifting, and inhibition, and we predicted that tDCS of the DLPFC may modulate these core executive function components simultaneously.

## Materials and Methods

### Participants

**Figure 1:**
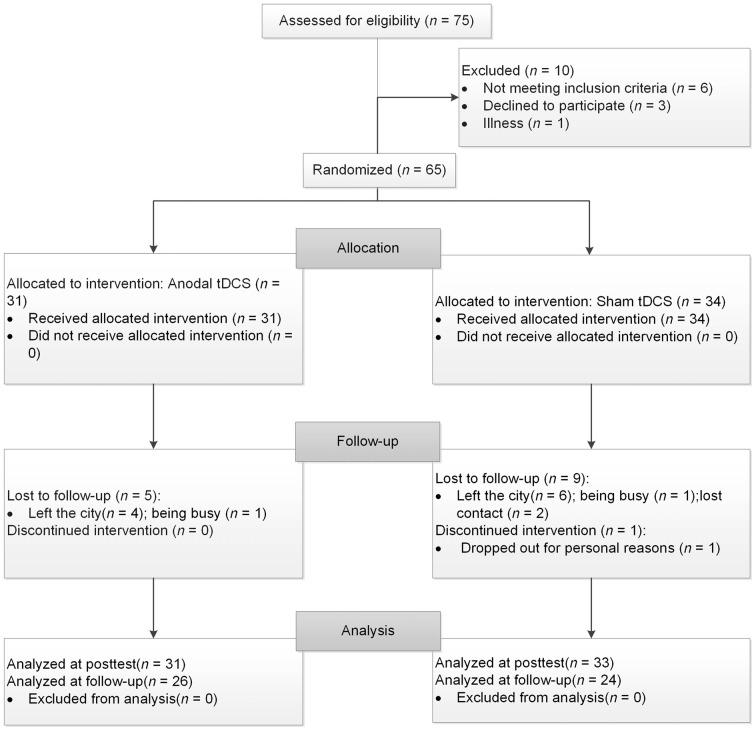
The flow chart of the study. tDCS, transcranial direct current stimulation.

Seventy-five healthy older volunteers from the community registered with this study. Participants met the following inclusion criteria: (1) age ≥60 years, (2) education ≥9 years, (3) normal global cognitive function (with a score ≥21 on the Montreal Cognitive Assessment-Beijing Version, MoCA-BJ; Yu et al., 2012), (4) a normal emotional state (with a score ≤16 on the Center for Epidemiologic Studies Depression Scale; CES-D; Roberts and Vernon, 1983), and (5) no history of neurological or psychiatric disorders or traumatic brain injury. After baseline evaluation, 10 participants were excluded from the study because they did not meet the inclusion criteria of cognitive function or emotional state (*n* = 6), they declined to participate for the long duration of the experiment (*n* = 3), or they became ill (*n* = 1). One participant dropped out after four sessions. Consequently, 64 participants aged 60–82 completed the study and their data were included in the final analysis, with 31 in the experimental group and 33 in the control group. Three months later, a total of 50 participants returned and finished the follow-up assessment, with 26 in the experimental group and 24 in the control group. Of the 14 drop-outs, 10 left the city, two were busy and two lost contact. Further information regarding the progression of participants through the trial is shown in Figure 1.

Participants signed informed consent documents before taking part in our experiment. This study was carried out in accordance with the recommendations of the ethics committee of the Institute of Psychology, Chinese Academy of Sciences. The protocol was approved by the The Code of Ethics of the World Medical Association (Declaration of Helsinki). All subjects gave written informed consent in accordance with the Declaration of Helsinki. In addition, the study was registered in the Chinese Clinical Trial Registry (ChiCTR) with the identifier ChiCTR-INR-16010036^1^.

### Procedure

A randomized clinical trial with single-blind and sham-controlled procedure was conducted. Participants were randomly divided into two treatment groups: an atDCS group and a sham tDCS group. They were unaware of the group assignments and study design. Block randomization with six participants per block was used. The randomization sequence was generated by a third party, who did not participate in this study, using an online randomization generation tool. An allocation number was used to maintain confidentiality before participants finished the baseline evaluation. The allocation ratio was 1:1.

All participants received 10 daily sessions (30 min/day) of tDCS stimulation. At baseline, they completed demographic questionnaires and a battery of neuropsychological tests. Of these neuropsychological tests, the digit span forward and digit span backward tasks (Gong, 1992) were used to measure working memory ability. The trail making test (Reitan, 1986) was used to measure comprehensive executive function. Additionally, participants completed some episodic memory tests, e.g., the source memory task and the verbal learning test. We did not analyze and report the memory data here because memory was not our concern. Before and after the tDCS treatment, three critical components of executive functioning were assessed using three computerized tasks: the two-back task for updating, the flanker task for inhibition, and the switching task for shifting. We controlled the interval between the pretest and posttest to be about a month, and there was no difference between the atDCS group (26 ± 6.5 days) and sham group (24.5 ± 5.7 days). Participants completed a brief post-study questionnaire (Fertonani et al., 2010) regarding their subjective experience in order to evaluate the adverse effects of stimulation. They rated the level of adverse feelings (i.e., itchiness, skin pain, heat, tingling, or other feelings) on a five-point Likert scale, with 1 indicating that they did not feel the described sensation at all and 5 indicating that they strongly felt the described sensation. They also evaluated the duration and the influence of these side effects on task performance.

To detect the long-term effect of stimulation, we invited all of the participants to return after 3 months of no contact. Only participants who completed all three executive function tasks were included in the analysis. Eventually, the data of 26 participants in the atDCS group and 24 in the sham group were collected. The average interval between the posttest sessions and follow-up sessions was 97.5 days (range = 84–122; standard deviation = 8.81). Again, no difference was found between the atDCS group (96.88 ± 9.94 days) and sham group (98.17 ± 7.34 days).

### Executive Function Measures

#### Two-Back Task

This task is commonly used to assess working memory updating. The stimuli were the digits one through nine. Participants were required to indicate whether the current digit was the same as the digit presented two trials beforehand. At the beginning of each block, a fixation cross was displayed for 1,000 ms. Then, the stimulus appeared for 500 ms, after which a blank screen was presented for 2,000 ms, during which participants made a response before the next stimulus appeared. The task consisted of five blocks, with each one containing 20 trials. The dependent variables for this task were the accuracy rate and reaction time (RT).

#### Flanker Task

The arrow version of the flanker task (Eriksen and Schultz, 1979) was used to measure inhibitory control. The stimuli were five arrows pointing to the right or the left, with the target arrow in the center and two flankers on each side of the target. Two types of trials randomly appeared: congruent trials, in which the flankers pointed in the same direction as the target arrow (<<<<< or >>>>>), and incongruent trials, in which the flankers pointed in the opposite direction to the target arrow (<< ><< or >><>>). Participants were required to press the right or left response button indicated by the direction of the target stimulus and to ignore the flankers. In each trial, the stimulus was presented until participants pressed a key or until 1,500 ms had passed, following a fixation cross (500 ms). After the stimulus, a blank screen appeared for 500 ms and the next trial began. There were a total of 80 formal trials. The primary index of inhibition was the interference score for the difference in the RTs between the incongruent trials and congruent trials.

#### Switching Task

**Figure 2:**
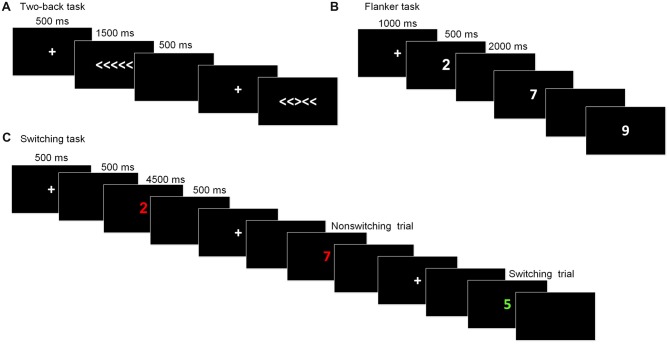
Schematic of the executive function paradigms. **(A)** Flanker task; **(B)** Two-back task; **(C)** Switching task.

The task-switching paradigm was used to measure shifting ability. The paradigm consisted of two tasks that appeared randomly and participants were required to switch between them: Task A involved categorizing a digit as odd/even and Task B involved categorizing a digit as smaller/larger than five. The stimuli were colored digits from one to nine (except for five) that were presented centrally. The color served as a task cue, with red for smaller/larger and green for odd/even. Each trial began with a fixation cross presented for 500 ms, followed by a blank screen for 500 ms. The stimuli appeared until participants made a response or 4,500 ms had elapsed. Then, a blank screen followed for 500 ms before a new trial started. Of the 80 formal trials, half were switching trials and the other half were repetition trials. The dependent variable for this task was the switching cost, which was calculated by subtracting the RT of the task-switch trials from those of the task-repetition trials. Prior to each task, instructions and practice sessions were given until participants fully understood the task and they were familiar with the task. The schematic presentations of the executive function tests were showed in Figure 2.

### Transcranial Direct Current Stimulation Protocol

A low constant current was delivered using a battery-driven stimulator, the DC-STIMULATION MC8 (NeuroConn, Munich, Germany), through a pair of 5 × 5 cm^2^ saline-soaked sponge electrodes. The target (anodal) electrode was placed on the scalp above the left DLPFC, corresponding to F3 in the 10-20 international electroencephalogram system (Jasper, 1958). The reference (cathodal) electrode was placed on the right deltoid muscle to avoid its possible inhibitory effect on the brain.

**Figure 3:**
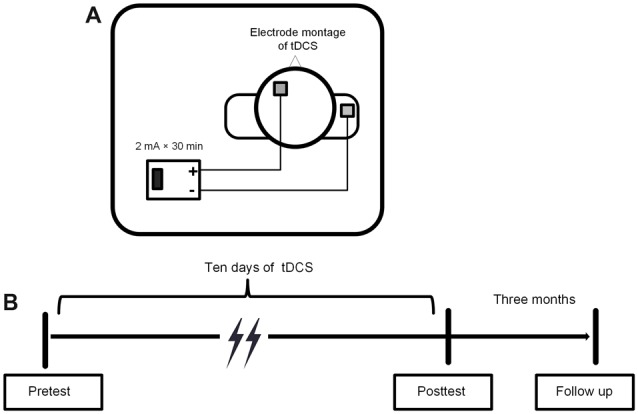
**(A)** Electrode montage of tDCS. **(B)** Experimental procedure of tDCS.

A constant current was delivered at an intensity of 2 mA for 30 min, with a 20 s ramp on and 20 s ramp off time at the beginning and end of the stimulation. The current density was 0.08 mA/cm^2^ and it was within safety limits (Nitsche et al., 2008; Bikson et al., 2016). For the sham stimulation, the electrodes were placed at the same position for 30 min, but the current lasted for 30 s at the beginning and end of the stimulation to produce a physical sensation that was identical to the active stimulation. The procedures of the study protocol and the tDCS electrode montage were showed in Figure 3.

### Data Analysis

The statistical analysis was conducted using IBM SPSS Statistics for Windows 21.0 (IBM Corporation, Armonk, NY, USA). Group differences in the baseline cognitive and demographic variables were examined using the independent-samples *t*-test and the chi-square test for the continuous and categorical data, respectively. The effects of the stimulation were examined using repeated-measures analyses of variance (ANOVAs), with group (atDCS vs. sham tDCS) as a between-subjects factor and test time (pre vs. post) as a within-subjects factor. The accuracy and RTs of the correct responses were the primary dependent variables. A significance level of *p* < 0.05 was set.

## Results

### Demographic and Clinical Characteristics

No significant differences were found between the two groups in terms of age, gender, or years of education (see Table 1 for demographic details). No differences were observed between the groups for global cognition, working memory, or executive function, as measured by the MoCA, digit span test and trail making test, respectively.

**Table 1:** Demographic characteristics and neuropsychological results (mean ± standard deviation).

	atDCS (*n* = 31)	Sham (*n* = 33)	*p*-value
Age (years)	66.55 ± 6.15	65.73 ± 3.70	0.52
Gender (female/male)	16/15	20/13	0.47
Education (years)	12.45 ± 2.38	12.12 ± 2.15	0.56
MoCA	26 ± 2.38	25.97 ± 2	0.96
Digit span forward	6.90 ± 1.56	6.94 ± 1.25	0.92
Digit span backward	5.03 ± 1.35	4.58 ± 1.25	0.17
Trail making test B-A	41.35 ± 35.88	30.18 ± 17.87	0.13

*MoCA, Montreal Cognitive Assessment*.

### Stimulation Effect

#### Two-Back Task

**Figure 4:**
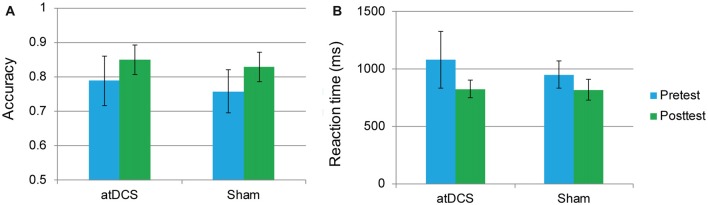
The two-back performance at pretest and posttest for the anodal transcranial direct current stimulation and sham stimulation groups. The plots display the mean accuracy rate **(A)** and mean reaction time (RT; **B**) in each group (anodal transcranial direct current stimulation and sham transcranial direct current stimulation) at pretest and posttest. Error bars represent standard errors of the mean. atDCS, anodal transcranial direct current stimulation.

An independent-samples *t*-test revealed that neither accuracy nor RT differed between the atDCS group and sham group (all *p*s > 0.05) at baseline. The repeated-measures ANOVA of groups (atDCS vs. sham tDCS) and test time (pre vs. post) revealed only a main effect of test time (accuracy: *F*_(1,62)_ = 24.01, *p* < 0.001, partial *η*^2^ = 0.279; RT: *F*_(1,62)_ = 21.61, *p* < 0.001, partial *η*^2^ = 0.258), with accuracy increasing and RT shortening significantly in both groups after stimulation. Importantly, the interaction between group and test time was not significant (accuracy: *F*_(1,62)_ = 1.12, *p* = 0.732; RT: *F*_(1,62)_ = 2.10, *p* = 0.152), revealing that, after stimulation, there was no greater stimulation gain in the two-back performance for the atDCS group compared with the sham group (Figures 4A,B).

#### Flanker Task

**Figure 5:**
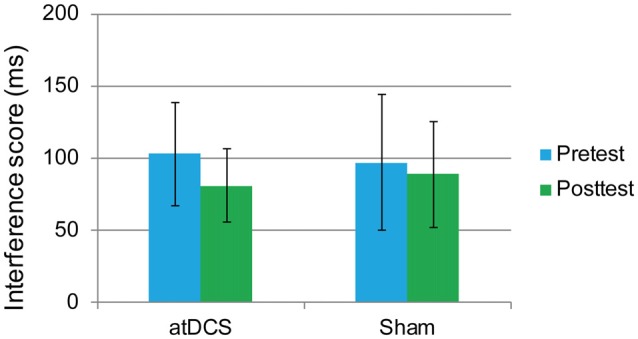
The plot displays the mean interference score of the RT in each group (anodal transcranial direct current stimulation and sham transcranial direct current stimulation) at pretest and posttest. Error bars represent standard errors of the mean. atDCS, anodal transcranial direct current stimulation.

In this task, the overall accuracy was high, with 97.40% at pretest and 98.71% at posttest. For this task, we were concerned only with the RT of the correct responses. The interference score (RT to incongruent trials minus RT to congruent trials) was used as the dependent variable. First, it was found that the difference between the atDCS group and the sham group was not significant before stimulation (*t*_(62)_ = 0.273, *p* = 0.786). Then, a repeated-measures ANOVA was conducted to measure the effects of the tDCS. The results revealed a main effect of test time (*F*_(1,62)_ = 4.37, *p* = 0.041, $\eta_{\text{p}}^{2}$ = 0.066) but no significant test time × group interaction (*F*_(1,62)_ = 0.88, *p* = 0.35; Figure 5), suggesting that the treatment did not affect the flanker task performance.

#### Switching Task

**Figure 6:**
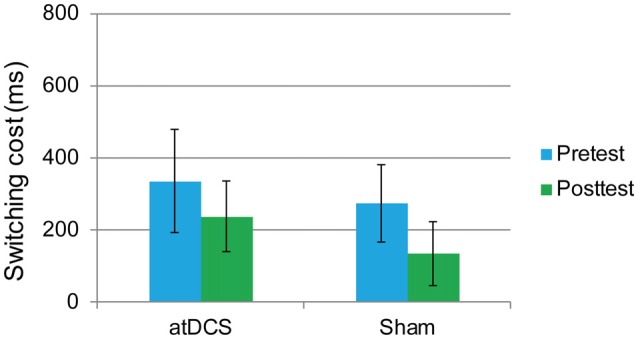
The plot displays the mean switching cost of the RT in each group (anodal transcranial direct current stimulation and sham transcranial direct current stimulation) at pretest and posttest. Error bars represent standard errors of the mean. atDCS, anodal transcranial direct current stimulation.

The performance of one participant was not recorded due to technical problems with the computer. The overall accuracy was 92.72% at pretest and 92.34% at posttest. We again focused on the RT of the correct responses. The dependent variable was the switching cost, which was calculated as the RTs in the switching trials minus the RTs in the repetition trials. At baseline, the switching cost was the same in the atDCS group and the sham group (*t*_(61)_ = 0.96, *p* = 0.34). Then, a repeated-measures ANOVA was conducted. The main effect of test time approached significance (*F*_(1,61)_ = 14.61, *p* < 0.001, partial *η*^2^ = 0.193), which may indicate a practice effect. Again, no significant interaction effects were observed (*F*_(1,61)_ = 0.45, *p* = 0.51). Switching cost was previously reported to be gender-dependent (Christakou et al., 2009). Therefore, to exclude possible gender effect in the performance of switching cost, we conducted subgroup analyses of repeated-measures ANOVA for male and female separately. Still, there were no significant test time × group interactions for male (*F*_(1,27)_ = 0.001, *p* = 0.98) or female (*F*_(1,32)_ = 0.504, *p* = 0.48), neither. Therefore, switching cost was not reduced by anodal stimulation (Figure 6).

### Follow-Up Effect

**Figure 7:**
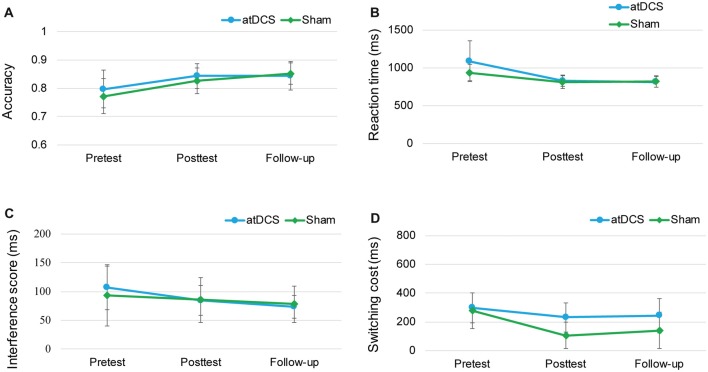
The plots display the mean accuracy rate **(A)** and mean RT **(B)** of the two-back task, the mean interference score of the flanker task **(C)** and the mean switching cost of the switching task **(D)** in each group (anodal transcranial direct current stimulation and sham transcranial direct current stimulation) at pretest, posttest and follow-up. Error bars represent standard errors of the mean. atDCS, anodal transcranial direct current stimulation.

Because of the delayed effects of tDCS on executive functioning demonstrated in previous studies (Doruk et al., 2014; Sandrini et al., 2014; Hsu et al., 2015b), we conducted the follow-up session to investigate whether the stimulation effect appeared over time. The repeated-measures ANOVAs with groups (atDCS vs. sham tDCS) and test time (pre vs. post vs. follow-up) revealed no significant effects for group or test time, and no interaction (all *ps* > 0.05) across the three tasks (Figure 7). The subgroup analysis for switching cost revealed that Test Time × Group interactions were not significant for either male (*F*_(2,38)_ = 0.916, *p* = 0.41) or female (*F*_(2,52)_ = 0.243, *p* = 0.79).

### Adverse Effects

All participants tolerated the stimulation well and no participants withdrew due to serious side effects. Each participant gave an average rating score for the strength, duration and influence of the physical feelings, which showed that there were no significant differences on average score between the two groups (atDCS group: 1.64 ± 0.31; sham tDCS group: 1.50 ± 0.21; *p* = 0.130 by independent-samples *t*-test).

## Discussion

In the last decade, tDCS has been proposed as a promising tool for delaying cognitive deterioration in healthy aging populations. The aim of the present randomized, sham-controlled study was to investigate whether tDCS with a multiple-days stimulation protocol would improve the executive function of healthy aging people, as assessed with the two-back task, flanker task and switching task paradigm. The atDCS and sham tDCS were applied on the scalp above the left DLPFC and task performance was then compared under these two stimulation conditions immediately after stimulation and 3 months after stimulation. To our knowledge, this is the first study to investigate the effect of multiple-sessions of tDCS alone on the executive function of healthy older adults.

Unfortunately, in the present study, we were unable to find statistical evidence for an anodal stimulation effect neither immediately or 3 months after the treatment, which may be ascribed to the absence of simultaneous cognitive training. Most previous studies (Park et al., 2014; Jones et al., 2015; Stephens and Berryhill, 2016) that combined cognitive training and a similar stimulation protocol demonstrated an improvement in working memory and transfer effects to the executive function of the elderly people. These studies focused on the augmentative effect of multiple sessions of stimulation on working memory training, demonstrating that training coupled with tDCS produced a larger effect than memory training alone. Meta-analytic evidence of healthy young populations has also shown that the combination of left DLPFC stimulation with working memory training, rather than stimulation alone, enhances working memory performance (Mancuso et al., 2016). Taken together, these findings suggest that, at least in the domain of executive function, tDCS may be an adjuvant to cognitive training rather than a cognitive modulator tool that independently has an effect. The cognitive enhancement potential of tDCS may occur through boosting the efficiency of training.

In addition, it is possible that anodal stimulation over the left DLPFC enhances the executive function performance independently of training, while some variables weaken the efficiency, causing null effects. A possible explanation is that tDCS benefits the executive function of particular groups of the population. For example, tDCS enhanced the performance of executive function on patients with psychiatric or neurological disorder, e.g., attention deficit/hyperactivity disorder (Nejati et al., 2017), Parkinson’s disease (Doruk et al., 2014; Swank et al., 2016). Maybe the worse executive function of patients at baseline brings more space for improvement. Furthermore, it has been revealed that less than 50% of participants have a physiological response to electric stimulation (López-Alonso et al., 2014; Wiethoff et al., 2014). Numerous inter-individual variables cause the heterogeneity on the efficacy of tDCS. First, gender (Chaieb et al., 2008; Fumagalli et al., 2010) is a possibly confusing factor. Chaieb et al. (2008) found that anodal tDCS heightened cortical excitability more significantly in the women subject group, comparing the male group. In addition, the executive function, especially switching cost, were previously reported to be gender-dependent (Christakou et al., 2009). However, in the present study, we have excluded the gender effect on switching cost by subgroup analyses. Second, aging might be another obstructive factor (Müller-Dahlhaus et al., 2008; Kishore et al., 2014). The decline of brain plasticity induced by aging prevents elderly people from benefitting from the cortical modulation by the stimulation. Certainly, we should take caution to conclude that the negative outcome is merely due to the age of the patients. Third, the stimulation effect is also mediated by gene polymorphism (Cheeran et al., 2008; Antal et al., 2010; Plewnia et al., 2013; Puri et al., 2015). As an example, in the study of Plewnia et al. (2013), the effect of tDCS on executive function was observed only in participants with COMT Met/Met homozygosity, which is related to higher dopaminergic activity in the prefrontal cortex. Except for age, gender and the genetic state, many of individual’s factors, such as education (Berryhill and Jones, 2012), head size, skull thickness, and so on, might also contribute to the various effects of tDCS. Given the small sample size used in the present study and typically used in previous tDCS literature (Brunoni et al., 2008; Tremblay et al., 2014; Medina and Cason, 2017; Westwood et al., 2017), individual differences, which could induce group differences in a small sample size, might be the exact reason why some stimulation protocols do not obtain effective results.

Another important aspect to take into account is the stimulation position. Functional neuroimaging studies have demonstrated that the three elemental components of executive function are predominantly executed by a fronto-parietal network or cognitive control network, mainly including the DLPFC (BA 9 and BA 46), frontopolar cortex, the orbitofrontal cortex, and anterior cingulate (3–6). Although the DLPFC is functionally relevant for processing executive function, multiple brain areas in the distributed network work collaboratively when processing executive functions. Thus, a greater effect is likely to be generated when simultaneously stimulating nodes in the functional networks instead of a single region. Consistent with this view, some researchers (Hill et al., 2016; Kim et al., 2016) suggested that to effectively modulate memory or working memory, multiple nodes of the memory network should be stimulated. More recently, high-definition tDCS (HD-tDCS) has provided a new method for stimulating multiple cortical sites. With more than two smaller electrodes and multi-channel stimulators, HD-tDCS delivers more focal stimulation than conventional tDCS (Gbadeyan et al., 2016).

Several limitations should be noted. First, we only recruited cognitively healthy older adults. It will become a limitation when generalizing our findings to other populations. Second, more control groups were absent. An experimental group with stimulation over the opposite polarity, i.e., anode over the right DLPFC, as well as a control group with cathode over the left arm, should be investigated. Lacking enough control groups, we should be careful to conclude a negative outcome. Finally, a relatively small sample size of older adults was used in our study, the negative results should be treated with caution.

In summary, this investigation showed that, relative to the sham tDCS, atDCS without cognitive training did not modulate the behavioral performance of the executive function in healthy older adults. Some implications from this study should be taken into account. First, to boost older adults’ executive function, combining cognitive training with brain stimulation is suggested in the future intervention studies. Second, due to the inter-individual variables, future studies could employ personalized stimulation protocols based on individual differences or use large sample sizes to eliminate individual differences. Third, stimulation over multiple sites that cover the specific functional network may be better than stimulation of a single site.

## Author Contributions

JuL conceived the idea. JuL and LH designed the study. LH analyzed the data and drafted the manuscript. ZZ assisted with data analysis and manuscript revision. JiL, WiW, WyW, XC and SC assisted with experiment implementation. JuL supervised the implementation, writing and revision of the manuscript.

## Conflict of Interest Statement

The authors declare that the research was conducted in the absence of any commercial or financial relationships that could be construed as a potential conflict of interest.

## Footnotes

^1^http://www.chictr.org.cn/index.aspx

## Abbreviations

ANOVA: analysis of variance
atDCS: anodal transcranial direct current stimulation
DLPFC: dorsolateral prefrontal cortex
HD-tDCS: high-definition transcranial direct current stimulation
RT: reaction time
tDCS: transcranial direct current stimulation.
